# A High Throughput Genetic Screen Identifies New Early Meiotic Recombination Functions in *Arabidopsis thaliana*

**DOI:** 10.1371/journal.pgen.1000654

**Published:** 2009-09-18

**Authors:** Arnaud De Muyt, Lucie Pereira, Daniel Vezon, Liudmila Chelysheva, Ghislaine Gendrot, Aurélie Chambon, Sandrine Lainé-Choinard, Georges Pelletier, Raphaël Mercier, Fabien Nogué, Mathilde Grelon

**Affiliations:** INRA de Versailles, Institut Jean-Pierre Bourgin, Station de Génétique et d'Amélioration des Plantes UR-254, Versailles, France; The University of North Carolina at Chapel Hill, United States of America

## Abstract

Meiotic recombination is initiated by the formation of numerous DNA double-strand breaks (DSBs) catalysed by the widely conserved Spo11 protein. In *Saccharomyces cerevisiae*, Spo11 requires nine other proteins for meiotic DSB formation; however, unlike Spo11, few of these are conserved across kingdoms. In order to investigate this recombination step in higher eukaryotes, we took advantage of a high-throughput meiotic mutant screen carried out in the model plant *Arabidopsis thaliana*. A collection of 55,000 mutant lines was screened, and *spo11*-like mutations, characterised by a drastic decrease in chiasma formation at metaphase I associated with an absence of synapsis at prophase, were selected. This screen led to the identification of two populations of mutants classified according to their recombination defects: mutants that repair meiotic DSBs using the sister chromatid such as *Atdmc1* or mutants that are unable to make DSBs like *Atspo11-1*. We found that in *Arabidopsis thaliana* at least four proteins are necessary for driving meiotic DSB repair via the homologous chromosomes. These include the previously characterised DMC1 and the Hop1-related ASY1 proteins, but also the meiotic specific cyclin SDS as well as the Hop2 *Arabidopsis* homologue *AHP2*. Analysing the mutants defective in DSB formation, we identified the previously characterised *AtSPO11-1*, *AtSPO11-2*, and *AtPRD1* as well as two new genes, *AtPRD2* and *AtPRD3*. Our data thus increase the number of proteins necessary for DSB formation in *Arabidopsis thaliana* to five. Unlike SPO11 and (to a minor extent) PRD1, these two new proteins are poorly conserved among species, suggesting that the DSB formation mechanism, but not its regulation, is conserved among eukaryotes.

## Author Summary

During fertilisation, paternal and maternal gametes meet to form the next generation zygote. The zygotic cells therefore contain two sets of chromosomes, paternal and maternal, called homologues. Gamete production depends on the completion of meiosis, during which the chromosome number is divided by two. For this to happen, homologous chromosomes associate into pairs called bivalents, where each chromosome is linked to its homologue by one or several chiasmata. These chiasmata reflect the formation of crossovers, one of the manifestations of the exchange of genetic material occurring during homologous recombination. Meiotic recombination is initiated by the formation of DNA double-strand breaks that are repaired using the homologous chromosome as a template, allowing the stable interactions between them. Although these events are strongly conserved among species, the molecular players are not. In order to investigate recombination in higher eukaryotes, we carried out a high-throughput mutant screen in the model plant *Arabidopsis thaliana*. The results obtained and presented in this paper describe the isolation and the characterisation of mutants defective in early steps of meiotic recombination from a collection of 55,000 lines, providing the first general view of the molecular players involved in early meiotic recombination in a higher eukaryote.

## Introduction

Organisms that reproduce sexually have acquired a specialized type of cell division, called meiosis, which allows the production of haploid gametes from a diploid mother cell. During prophase I of meiosis, homologous recombination occurs and can lead to the reciprocal exchange of genetic material between homologous chromosomes also called crossovers (COs). COs establish physical links between homologous chromosomes, allowing their correct segregation during the first meiotic division.

In budding yeast, meiotic recombination is initiated by the formation of numerous programmed double strand breaks (DSBs) catalysed by the Spo11 protein [1],[2]. Spo11 is evolutionarily conserved among different kingdoms with homologues in animals, fungi, plants and protists [1],[3]. In most species, Spo11 is encoded by a single gene and its disruption leads to sterility and meiotic recombination defects, suggesting that the catalytic activity of Spo11 is also conserved [4]–[7]. The *Arabidopsis* genome and also genomes of species in some other eukaryotic lineages [3] contains several *Spo11* homologues, *AtSPO11-1*, *AtSPO11-2* and *AtSPO11-3* [8]–[13]. Functional data obtained in *Arabidopsis* showed that *AtSPO11-1* and *AtSPO11-2* are required for meiotic recombination [8],[14],[15] whereas *AtSPO11-3* encodes a topoisomerase VI A subunit involved in somatic endoreduplication [9],[12].

To catalyse meiotic DSBs in *S. cerevisiae*, the Spo11 protein does not act alone and needs at least nine additional proteins (Rad50, Mre11, Xrs2, Rec102, Rec104, Rec114, Ski8, Mer2 and Mei4) [1],[2]. Furthermore, the kinase activity of Cdc7 and Cdc28 directly regulates DSB formation via phosphorylation of the Mer2 protein [16]–[18]. Analysis of the complete genomic sequences now available for numerous species showed that few of these *S. cerevisiae* “DSB proteins” are conserved in other kingdoms [1],[2]. Besides, even when protein sequences were conserved, functional divergences were often observed. For example, orthologues of Rad50, Mre11, and Xrs2 are not required for DSB formation in *S. pombe*, *C. cinereus*, or *A. thaliana*, although they are required for processing of meiotic DSBs [19]–[24]. Likewise, the Ski8 orthologue is required for DSB formation in *S. cerevisiae*, *S. pombe*, and *S. macrospora*, but not in *A. thaliana* [25]–[28]. In *S. pombe*, the “DSB proteins” Mde2, Rec6, Rec10, Rec15 and Rec24 were isolated in a genetic screen for meiotic recombination or by a systematic knock-out of genes which were up-regulated during meiosis [29]–[32]. Similarly to the situation in *S. cerevisiae*, no clear homologues of these “DSB proteins” can be identified in organisms other than those closely related.

As a consequence, no overall view of the DSB-forming proteins is available in higher eukaryotes, and only a few players of this recombination step have been described. Mei-P22, for example, was isolated in a large screen for P-element insertions in *D. melanogaster* [33],[34]. The *Mei-P22* mutant shows a drastic decrease in γ-H2Av foci (marker of DSBs) which can be partially restored by X-Ray treatment indicating that Mei-P22 is required for DSB formation [35]. The *Arabidopsis* protein AtPRD1 (for *Arabidopsis thaliana* Putative Recombination initiation Defects 1) and its mouse homologue Mei1 were found by phenotype-based screens for mutations causing infertility as a consequence of meiotic defects [36]–[38] and both were found to be necessary for meiotic recombination initiation [36],[37],[39].

We therefore screened a saturated collection of *Arabidopsis thaliana* mutants with the aim of isolating *Atspo11-like* mutants. With this screen, we successfully identified two types of genes according to their function during meiotic recombination: mutants that repair DSBs using sister chromatids such as *Atdmc1* [40] or mutants that are unable to make DSBs like *Atspo11-1* [8]. In the latter population, we identified two new DSB-forming proteins, AtPRD2 and AtPRD3, and thus increased the number of proteins necessary for DSB formation in *Arabidopsis* to five, including AtSPO11-1, AtSPO11-2 and AtPRD1. In addition, we provide new data concerning the *Arabidopsis* Hop2 homologue (AHP2, [41]) and the meiosis specific cyclin SDS [42] by demonstrating that these proteins are required for homologous chromosome driven DSB repair, probably through a DMC1 dependent pathway.

## Results

### A genome-wide screen for genes required for early meiotic recombination

**Figure 1:**
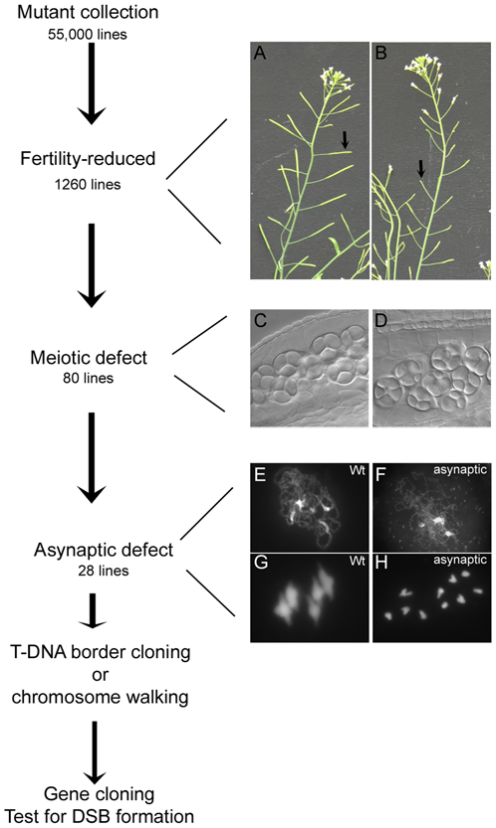
Screening procedure. A mutant collection of 55,000 lines was screened for fertility defects on the basis of reduced silique elongation (arrows, compare: (A) wild type to (B) a low fertility line). In a second step, DIC microscopy on developing pollen mother cells was used to analyse male meiotic products. While normal meiosis produces four haploid cells encased in a single callose wall forming a tetrad of microspores (C), meiotic defects lead to the generation of irregular tetrads or polyads (D). DAPI staining of male meiocytes was then undertaken on meiotic mutants (E–H). In wild type, homologous chromosomes synapse at prophase (E) and five bivalents are easily observed at metaphase I (G). Mutant lines showing synapsis defects (F) and a shortage of chiasma at metaphase I (H) were selected. For each of these, genetic and molecular analyses allowed the construction of complementation groups and the cloning of the mutated genes. For each complementation group, a test for meiotic DSB formation was performed.

Mutants defective in meiotic DSB formation have been described in *Arabidopsis thaliana* [8],[15],[36]. They display a typical “asynaptic” phenotype, characterised by an absence of synapsis during prophase I and a drastic reduction in chiasmata at metaphase I (Figure 1E–1H). In order to identify the entire set of proteins necessary for meiotic recombination initiation, we set up the screen presented in Figure 1. Firstly, a saturated *Arabidopsis thaliana* mutant collection (available at http://www-ijpb.versailles.inra.fr/en/sgap/equipes/variabilite/crg/) was screened for fertility defects. While wild-type *Arabidopsis* plants reproduce by self-fertilisation to produce 50–70 seeds per fruit (silique, arrow Figure 1A), reproduction defects are associated with seed set decrease, and can be scored on the basis of short siliques (Figure 1B, arrow). Next, differential interference contrast (DIC) observation of male meiotic products in the low fertility lines was undertaken to identify those impaired at meiosis. In wild type, the four male meiotic products forming a tetrahedric structure encased in a single callose wall can be observed just after meiosis (Figure 1C), whereas meiotic defects result in irregular tetrads or polyads containing a variable number of daughter cells (Figure 1D). Following this step, we recovered 80 meiotic mutants out of the 55,000 lines of the collection. We then further investigated the meiotic defects of the selected lines by DAPI staining after chromosome spreading (Figure 1E–1H), and found 28 mutant lines showing the typical “asynaptic” phenotype we were looking for (Table 1).

**Table 1:** Screen results and allelic series studied.

Gene name MIPS	Line	Allele number	Ecotype	Chiasmata per cell	Nature of the mutation
	Wild type		Ws-4	7.4	
	Wild type		Col-0	9.2	
***AtSPO11-1***	DYK209	*Atspo11-1-1*	Ws-4	0.5 (n = 30)	T-DNA tagged [8]
At3g13170	EYL111		Ws-4	nd	T-DNA tagged
	E24*	*Atspo11-1-2*	Col-0	0.07 (n = 14)	1 bp change [8]
***AtSPO11-2***	FAT2		Ws-4	0 (n = 7)	29 bp deleted
At1g63990	FAE57		Ws-4	0 (n = 71)	nd
***AtPRD1***	EEO4	*Atprd1-1*	Ws-4	0 (n = 49)	T-DNA tagged [36]
At4g14180	EFD91		Ws-4	0 (n = 21)	12 bp deleted
***AtPRD2***	E19-68*	*Atprd2-1*	Col-0	0.04 (n = 51)	1 bp change G->A (early stop)
At5g57880	EKT10	*Atprd2-2*	Ws-4	0 (n = 53)	1 bp change G->C (splicing site)
	EAN68	*Atprd2-3*	Ws-4	0 (n = 63)	T-DNA tagged
	EXX73	*Atprd2-4*	Ws-4	0 (n = 23)	whole gene deleted
	FCE21	*Atprd2-5*	Ws-4	0 (n = 40)	1 bp added (early stop)
*AtPRD3*	EFB36	*Atprd3-1*	Ws-4	0 (n = 59)	1 bp change C->T (early stop)
At1g01690	ECH20	*Atprd3-2*	Ws-4	0 (n = 81)	7 bp deleted (early stop)
	GABI677D06*	*Atprd3-3*	Col-0	0 (n = 39)	T-DNA tagged
	GABI357H01*	*Atprd3-4*	Col-0	0.04 (n = 79)	T-DNA tagged
	DZT29	*Atprd3-5*	Ws-4	0 (n = 19)	5′ deletion
***AHP2***	EYU48	*ahp2-2*	Ws-4	0.27 (n = 45)	5′UTR deletion
At1g13330	EXI5	*ahp2-3*	Ws-4	nd	nd
***AtDMC1***	EXO2		Ws-4	0 (n = 25)	nd
At3g22880	EGO28		Ws-4	nd	T-DNA tagged
	Line3668*	*Atdmc1-1*	Ws	0.04 (n = 52)	T-DNA tagged [40]
***ASY1***	EJO3		Ws-4	1.5 (n = 44)	1 bp change (T->G) (early stop)
At1g67370	EJZ8	*asy1-3*	Ws-4	1.5 (n = 92)	T-DNA tagged
	Salk_144182*	*asy1-2*	Col-0	1.4**	T-DNA tagged
***SDS***	EGS85		Ws-4	0.07 (n = 15)	4 bp deleted (early stop)
At1g14750	EGS481		Ws-4	0.2 (n = 15)	3′ deletion
	EVM123		Ws-4	0 (n = 41)	5′ deletion
	FAG105	*sds-3*	Ws-4	0.3 (n = 74)	T-DNA tagged
	SAIL129F09*	*sds-2*	Col-0	0.1 (n = 41)	T-DNA tagged
	DMC6		Ws-4		translocations
	FBW201		Ws-4		
	EGX128		Ws-4		
	EDA42		Ws-4		
	EFC59		Ws-4		

The 28 mutant lines (Ws-4 accession) isolated in this study are presented as well as additional alleles (asterisk) obtained in another accession (Col-0). Each of the mutations is presented within its complementation group together with the name of the mutated gene. Chiasma count at metaphase I is provided as well as the nature of the mutation. Full sequence information is provided in Table S1. ** Described in [50], nd: not determined, n: number of cells.

The mutant collection used in this study was generated by T-DNA transformation [43]. Genetic analyses were therefore used to identify mutations that were tagged by the T-DNA insert, which was the case for seven out of the 28 mutants. For these, the mutated genes were recovered by T-DNA border isolation and sequencing (see Materials and Methods). In order to identify the genes in cases where the mutations were not linked to the T-DNA, the semi-sterile mutant plants (Ws-4 background) were crossed to wild-type Col-0 accession. For each F2 populations, 31 mutant plants were selected and genotyped using SNPlex technology (Applied Biosystems, http://www.appliedbiosystems.com) for 48 Ws-4/Col-0 polymorphic markers spanning the whole genome (Text S1). This rough mapping allowed the quick identification of putative allelic mutations. These were then tested by a direct complementation test with referenced mutations or by candidate gene sequencing (see Materials and Methods). For loci that did not colocalise with a putative candidate gene, fine gene mapping was carried out using additional semi-sterile plants that were genotyped for microsatellite markers in the selected genomic region.

Taken together, all these results allowed 9 complementation groups to be defined, which contained 2 to 4 alleles (Table 1 and Table S1). Five mutations could not be cloned because they were found to map with two unlinked regions of the genome, suggesting that a major translocation had occurred, probably as a consequence of the T-DNA mutagenesis. These lines were not investigated further (Table 1).

Among the 9 loci identified, some corresponded to previously identified genes: *AtSPO11-1* [8], *AtSPO11-2* [15], *AtPRD1* [36], *AtDMC1* [40], *ASY1* [44], *SDS* [42], and *AHP2* [41]. Two loci, however, represent uncharacterised genes and were therefore named *AtPRD2* and *AtPRD3* for *Arabidopsis thaliana* Putative Recombination initiation Defects 2–3.

### Identification of proteins required for DSB formation *versus* proteins necessary for IH-DSB repair

Among the 9 loci identified in this study, *AtSPO11-1*, *AtSPO11-2* and *AtPRD1* were previously shown to be necessary for meiotic DSB formation [8],[15],[36]. This is not the case, however, for *AtDMC1*, also identified in our screen. In the *Atdmc1* mutant, meiotic DSBs are formed as in wild type but repaired via a RAD51 dependent pathway so that chiasmata are not formed, which explains the presence of ten intact univalents at metaphase I instead of five bivalents [36],[40],[45]. As Dmc1 is one of the major players involved in inter-homologue (IH) bias observed during meiotic recombination, it is very likely that in *Atdmc1* mutants, repair occurs using the sister chromatid rather than the homologous chromosome as the template [40],[46].

**Figure 2:**
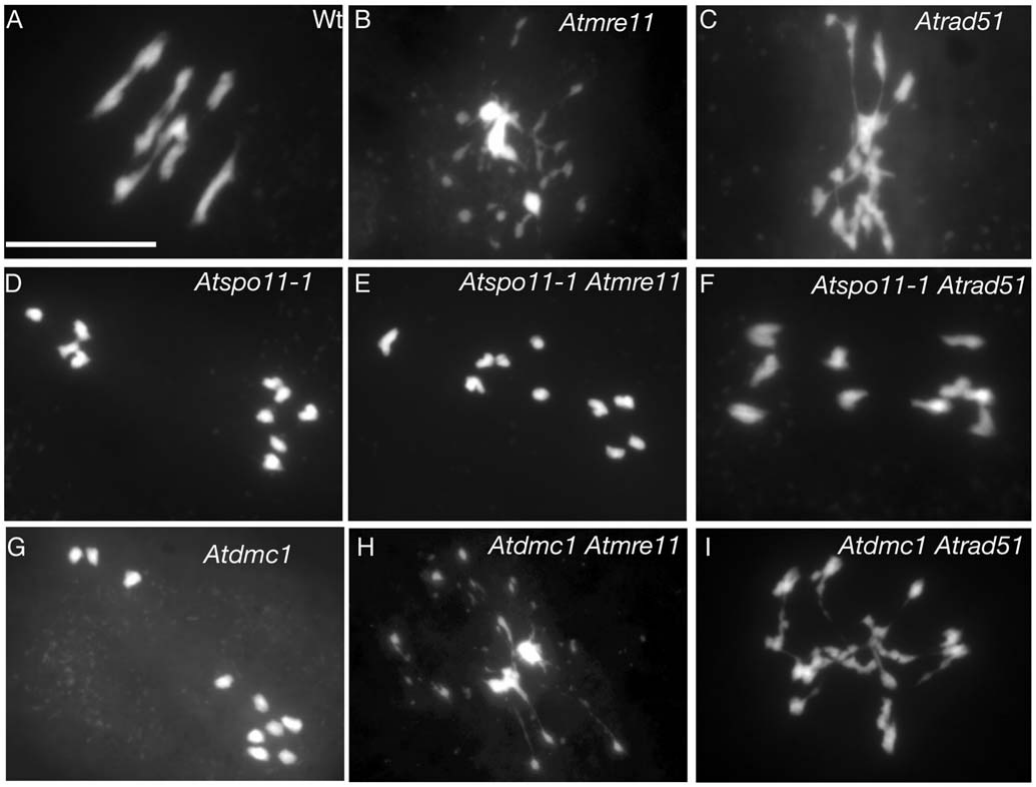
Test for meiotic DSB formation in the asynaptic mutants. In wild-type pollen mother cells, the ten *Arabidopsis* chromosomes are associated into five bivalents. Anaphase I in wild type (A) separates the homologous chromosomes in two equilibrated groups while *Atmre11* (*Atmre11-3* in B) and *Atrad51* (*Atrad51-1* in C) mutants show severe chromosome fragmentation at the first meiotic division. In *Aspo11-1* or *Admc1* mutants, ten intact univalents are observed at metaphase I that segregate randomly at anaphase I (shown for *Atspo11-1-1* in D and *Atdmc1-1* in G). DNA fragmentation of *Atrad51* and *Atmre11* is abolished in an *Atspo11-1* mutant background (shown for *Atspo11-1-1Atmre11-3* in E and for *Atspo11-1-1Atrad51-1* in F) but not in an *Atdmc1* context (*Atdmc1Atmre11-3* in H and *Atdmc1Atrad51* in I). Scale bar, 10 µm.

**Figure 3:**
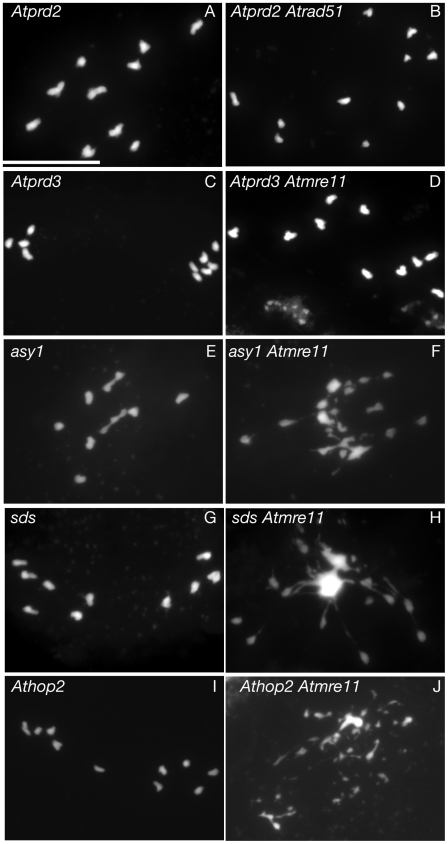
Mutations in *AtPRD2* and *AtPRD3* but not in *ASY1*, *SDS*, or *AHP2* abolish DSB repair defects. All pictures represent DAPI-stained male meiocytes at the metaphase I/anaphase I transition. (A) *Atprd2-1*, (B) *Atprd2-1Atrad51-1* double mutant, (C) *Atprd3*, (D) *Atprd3-1Atmre11-3*, (E) *asy1-3*, (F) *asy1-3mre11-3*, (G) *sds-2*, (H) *sds-2Atmre11-3*, (I) *Athop2-2/ahp2-2*, (J) *Athop2-2Atmre11-3*. Scale bar, 10 µm.

In order to discriminate between these two situations (DSB formation defects or IH-bias repair defects) we introgressed each of the isolated “asynaptic” mutations into mutants defective for meiotic DSB repair such as *Atmre11* or *Atrad51*. As shown previously, in both the *Atmre11-3* and *Atrad51-1* mutants, DSBs are formed but abnormally processed, leading to pronounced chromosome fragmentation during anaphase I ([22],[47] and Figure 2B and 2C). This DNA fragmentation was abolished in *Atspo11-1Atmre11-3* and *Atspo11-1Atrad51-1* double mutants ([22],[47]; Figure 2E and 2F) whereas it persisted in both *Atdmc1Atmre11-3* Figure 2H) and *Atdmc1Atrad51-1* ([36],[45]; Figure 2I), demonstrating that DSBs are absent in *Atspo11-1* but present in the *Atdmc1* mutant. As shown in Figure 3, chromosome fragmentation in *Atmre11* or *Atrad51* was also alleviated by of *Atprd2-1* or *Atprd3-1* mutations but not by crosses with the *asy1*, *sds* or *ahp2-2/hop2-2* mutants. Thus, our results show that the screen we conducted identified two functional families.

**Figure 4:**
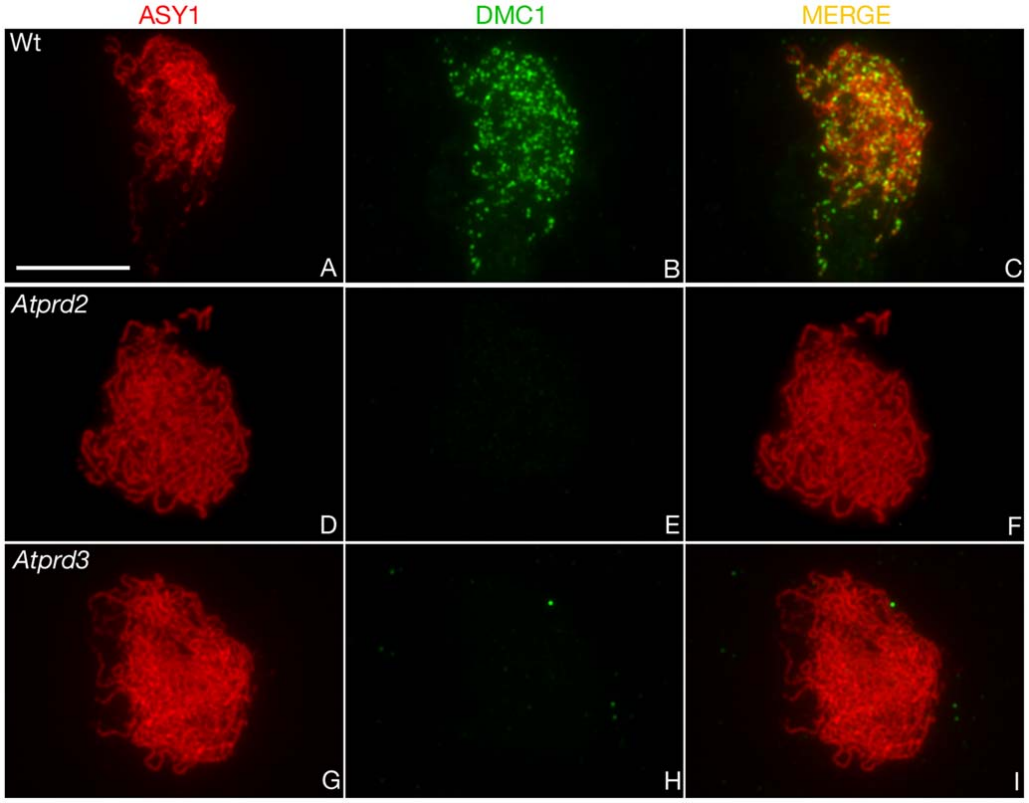
The *Atprd2* and *Atprd3* mutants are defective in DMC1 foci formation. Immunolocalisation of ASY1 and DMC1 in wild-type (A–C), *Atprd2-1* (D–F) and *Atprd3-1* (G–I) male meiocytes. For each cell, the ASY1 signal (red), DMC1 signal (green) and overlay (MERGE) are shown. Scale bar, 10 µm.

The first family contains AtSPO11-1, AtSPO11-2, AtPRD1, AtPRD2 and AtPRD3, all essential for meiotic DSB formation. To confirm that *Atprd2* and *Atprd3* were defective in early recombination processes, we analysed the nuclear distribution of the DMC1 protein. Its appearance on meiotic chromosomes during prophase is thought to reflect the progression of recombination repair [48]. Dual immunolocalisation of ASY1 and DMC1 was performed to follow the dynamics of recombination during early prophase I of meiosis. DMC1 foci appear at the leptotene stage of meiosis ([49]; Figure 4) and then decrease throughout meiotic prophase I until they completely disappear at pachytene stage (not shown). DSB deficient mutants *Atspo11-1* and *Atprd1* show a total absence of DMC1 foci indicating that localisation of recombinase DMC1 onto chromosomes is dependent upon DSB formation [36]. Likewise, DMC1 staining was completely missing in *Atprd2-1* (Figure 4D–4F) and *Atprd3-1* mutants (Figure 4G–4I). Taken together these data show that no meiotic DSBs are formed in *Atprd2* and *Atprd3* mutants.

The second functional class isolated in this screen contains *AtDMC1*, *ASY1*, *AtHOP2*/*AHP2* and *SDS*. Our study shows that all four are required for normal levels of chiasma formation and that this is not a consequence of a major defect in the level of meiotic DSB. However, although a weak effect on the meiotic DSB level would probably not have been detected in our experiments and cannot be ruled out, it could certainly not explain the major CO level decrease observed. Since absolutely no trace of chromosome fragmentation was observed associated with the shortage of chiasma in any of the isolated alleles, DSB repair must occur very efficiently in these backgrounds.

### The meiosis specific cyclin SDS is required for DMC1-mediated DSB repair using the homologous chromosome

*SDS* encodes a meiotic cyclin-like protein, distinct from all other known *Arabidopsis* cyclins [42]. Here we found that SDS is not required for meiotic DSB formation but is necessary for meiotic DSB repair via the homologous chromosome. In the absence of *SDS*, DSB repair acts efficiently (since chromosome fragmentation was never observed) but probably onto the sister chromatid, explaining the absence of CO formation. A similar phenotype was previously described for the *Atdmc1* mutation and, to a lesser extent, for *asy1* ([40],[50], see discussion). Our data therefore suggest that SDS could represent a new factor necessary for meiotic DMC1 driven homologous chromosome bias. In order to test this hypothesis, we analysed DMC1 and RAD51 foci formation in this background.

**Figure 5:**
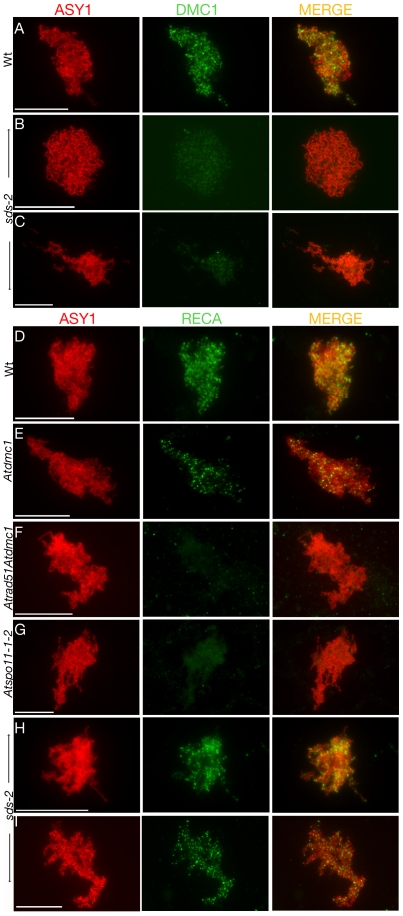
Co-immunolocalisation of the *Arabidopsis* recombinases with ASY1 in male meiocytes. Immunolocalisation of ASY1, DMC1, or DMC1/RAD51 (RECA) and overlay (MERGE) in wild-type (Wt A, D), *sds-2* (B–C, H–I), *Atdmc1* (E), *Atrad51Atdmc1* (F), and *Atspo11-1* (G) male meiocytes. Scale bar, 10 µm.

We used antibodies that recognise either AtDMC1 [36],[49] or both AtRAD51 and AtDMC1 (RECA-like, originally raised against the tomato RAD51 protein, [51]). We verified that when we used these anti-RECA like antibodies, foci were still observed in both *Atdmc1* (Figure 5D) or *Atrad51-1* (not shown), but had completely disappeared in *Atdmc1Atrad51-1* (Figure 5F), confirming that these antibodies recognize both AtDMC1 and AtRAD51 proteins. Both AtDMC1 and RECA-like foci disappear completely in the DSB defective *Atspo11-1* mutant (Figure 5G and [36]), confirming that they represent sites where DSB are initiated. In the present study, results with both the antibodies in the wild type were very similar to those of the previous studies [36],[49],[51],[52]. Numerous foci appeared at leptotene (Figure 5A and 5D) and decreased progressively until mostly disappeared at pachytene (not shown).

Interestingly, the behaviour of DMC1 localisation in the *sds-2* mutant was completely different to that of wild type. No DMC1 chromatin associated foci were observed in *sds* mutant cells (Figure 5B and 5C), whereas RECA-like foci still remained (Figure 5H and 5I), suggesting that mislocalisation of AtDMC1, but not of AtRAD51, is responsible for the recombination defects observed in this mutant.

### Characterisation of the two new DSB–forming proteins, AtPRD2 and AtPRD3

*Atprd2* and *Atprd3* mutants did not show any vegetative growth defects, but showed reduced silique elongation, indicative of fertility defects (Figure S1). The sterility of *Atprd2* and *Atprd3* mutants was correlated with abortion of the male and female gametophytes (Figure S2). Furthermore, examination of earlier stages of male gametophyte development revealed aberrant meiotic products (Figure S2) correlated with synapsis failure and a total absence of chiasmata as detailed in Figure S3.

**Figure 6:**
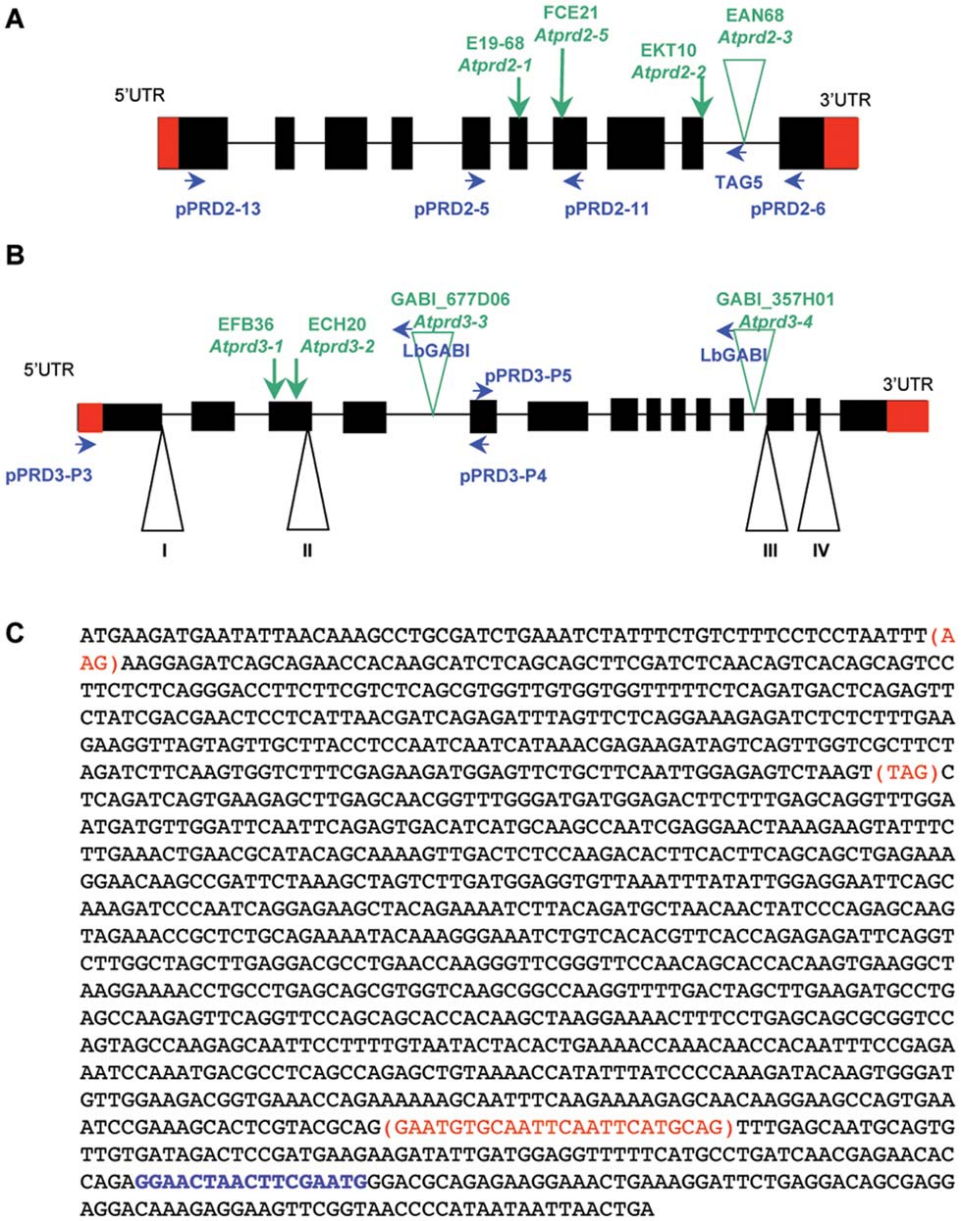
The *AtPRD2* and *AtPRD3* exon/intron structure. (A,B) Schematic representation of the *AtPRD2* and *AtPRD3* coding sequences. Exons are represented as black boxes and the UTR in red. Mutations are indicated in green, and primers used for genotyping in blue. The intron/exon junctions subjected to alternate splicing are indicated by numbered triangles. (C) The *At1g01690/AtPRD3* predicted cDNA sequence according to NCBI is shown in black. Additional nucleotides found in splicing variants I, II, or III are indicated in red. Nucleotides deleted in splicing variant IV are indicated in blue. In the Col-0 ecotype, among nine cDNA clones, three showed modification I, four modification II, and two modifications I and III. From the Ws cDNA, six clones had the reference sequence while 5 only had modification I and one had modifications I and IV.

**Figure 7:**
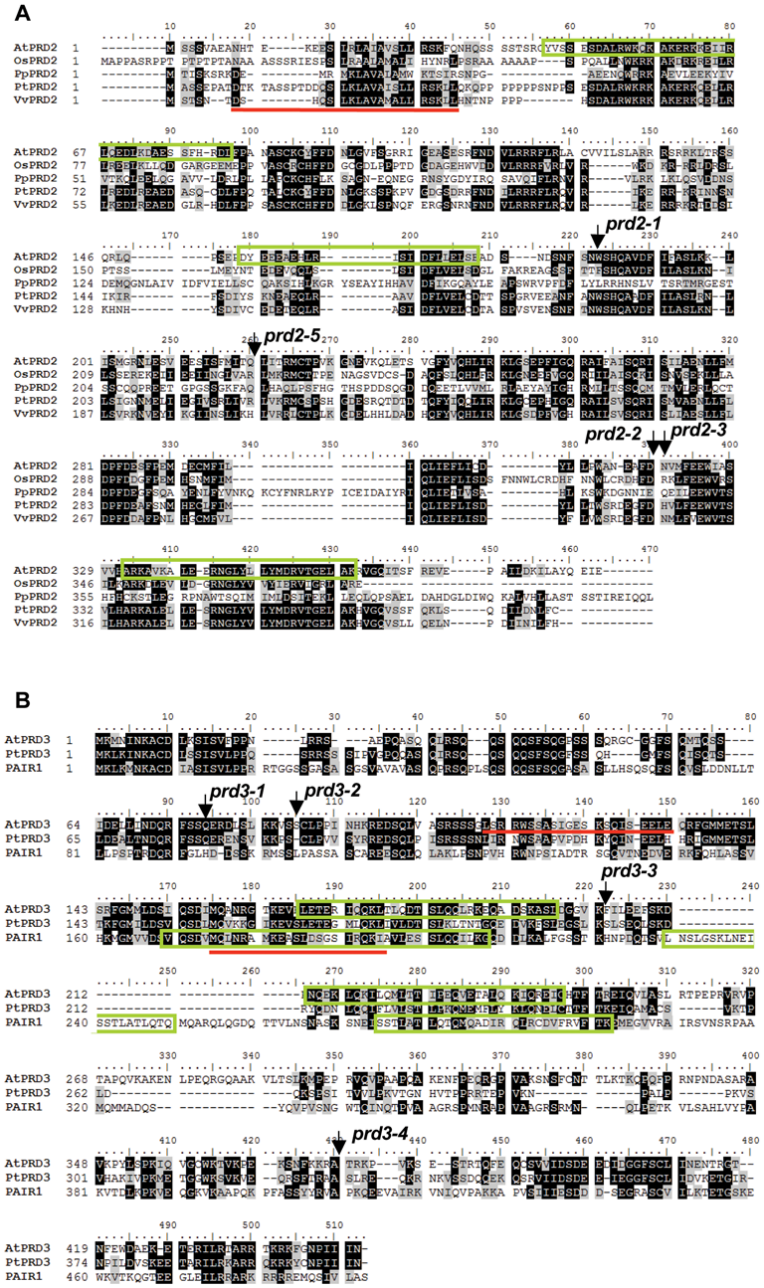
The PRD2 and PRD3 protein families. Protein sequence alignment of PRD2 (A) and PRD3/PAIR1 homologues (B). Os: *Oryza sativa*, Pp: *Physcomitrella patens*, Pt: *Populus tricocarpa*, Vv : *Vitis vinifera*; Numbers indicate amino acid position. Identical amino acids are indicated by a black box, similar amino acids by a grey box. Predicted coiled-coil structures are shown by a green box and helix-turn-helix motifs are underlined in red. Mutations in the studied alleles are indicated.

The *AtPRD2* gene (At5g57880) is 2119 bp long, contains ten exons and nine introns (Accession number FN356233 EMBL-EBI, Figure 6A) and encodes a protein of 385 amino acids (aa, molecular weight 44 kDa, pKi 5.5) with no obvious functional domains (Figure 7A). Network protein analysis (http://www.predictprotein.org/ and http://npsa-pbil.ibcp.fr/) predicted the presence of several α-helices throughout the protein and three coiled-coil motifs in the middle of the protein (aa 43 to 82, aa 154 to 181 and aa 332–359), suggesting that AtPRD2 is a globular protein [53]. We also observed several S/T-P-X-X and S/T-S/T-X-X motifs and a helix-turn-helix motif (10 to 31 aa) (http://npsa-pbil.ibcp.fr/), which are abundant in many DNA-binding proteins [54]. Database searches, using the BLASTP program (Blosum 45), for proteins similar to AtPRD2 produced the highest scores for proteins from other plant species such as *Oryza sativa*, *Populous trichocarpa*, *Vitis vinifera* and *Physcomitrella patens* (for accession numbers see Materials and Methods). A multiple sequence alignment of AtPRD2 homologues (Bioedit software version 7.0.9.0) revealed overall sequence conservation over the entire length of the protein (Figure 7A). However, despite using several PSI-blast iterations (Blosum 45) with AtPRD2 and its plant homologues, no significant similarities were found with proteins outside the plant kingdom.

The *AtPRD3* gene (At1g01690) is predicted to contain 14 exons, encoding a predicted 1350 bp cDNA for a 449 aa protein (50 kDa, pKi: 10.3). Isolation and sequencing of the full-length *AtPRD3* cDNA from flower buds of both Ws and Col-0 accessions, however, identified different PCR products (Figure 6B). Moreover, the differences we observed between the predicted cDNA sequence and the sequence of the PCR products from *AtPRD3* cDNA were at the exon/intron junctions, suggesting that the different cDNA products arose from alternative splicing. These splicing variants are likely to be functional given that the open reading frame is preserved in all the variants sequenced. However, further experiments will be needed to determine whether these variations play a regulatory role. BlastP analysis (Blosum 45) found a significant level of similarity (29% identity and 46% similarity) with the rice meiosis protein PAIR1 sequence (homologous pairing aberration in rice 1) [55]. The rice *pair1* mutant exhibits a marked defect in synapsis associated with a total loss of meiotic chiasmata [55]. This is similar to the phenotype observed in *Arabidopsis prd3*, suggesting that *AtPRD3* and *OsPAIR1* are orthologues. Sequence similarities were also observed with proteins from other plant species, but not in the basal plant *Physcomitrella patens*. No AtPRD3 homologues were found outside plants using PSI-BLAST analysis (Blosum 45). Multiple alignment of AtPRD3 homologues revealed the overall conservation of these proteins, with the largest number of conserved residues in the N-terminal section of AtPRD3 (Figure 6B).

Structural analysis of AtPRD3 and OsPAIR1 proteins failed to reveal any recognizable domains, but we did observe an abundance of α-helixes and several coiled-coil motifs in the centre of the protein (Figure 7B and [55]).

## Discussion

### Screen overview

In budding yeast, initiation of meiotic recombination is catalysed by the Spo11 protein but it is also dependent on numerous additional proteins [1]. Moreover, the behaviour of these proteins (immunolocalisation and physical interactions) revealed that they are interconnected [17], [25], [56]–[59], suggesting that DSB formation is mediated by a large protein complex. Nevertheless, the existence of a “DSB complex” in higher eukaryotes remains completely hypothetical since the majority of budding yeast “DSB proteins” are not conserved.

We performed a large scale genetic screen to identify meiotic mutants among 55,000 *Arabidopsis* T-DNA mutant lines. Eighty mutants affected in meiosis were recovered and 28 showed synapsis defects associated with a drastic reduction in chiasmata formation, defined as a “*spo11*-like” class of mutants. Meiotic genes responsible for the mutations that were cloned were found to be distributed in nine loci. Therefore we can conclude from this screen that at least nine genes are required for normal levels of CO and synapsis in *Arabidopsis thaliana*. Nevertheless, this number may still be underestimated since genes present as multicopies in the genome are not isolated by T-DNA mutagenesis.

### At least five proteins are necessary for DSB formation in *Arabidopsis thaliana*

Previous studies demonstrated that AtSPO11-1, AtSPO11-2 and AtPRD1 are required for meiotic DNA DSB formation in *Arabidopsis* [14],[22],[36],[47]. Here, we showed that AtPRD2 and AtPRD3 belong to this same functional family. Thus we now know that at least five proteins are absolutely required for DSB formation in *Arabidopsis*. In budding yeast, nine proteins in addition to Spo11 are essential for DSB formation, and in *S. pombe* six have been described (the ScRec114 orthologue Rec7, the Ski8 orthologue Rec14, Mde2, Rec6, Rec15, and Rec24, [30],[31],[60]) indicating that DSB formation is a complex mechanism which requires the function of numerous proteins.

AtPRD3 is a protein with no recognizable domains and appears to be the orthologue of the -rice PAIR1 [55]. Structural analysis of rice PAIR1 and AtPRD3 shows a cluster of α-helical coiled-coil motifs that are often present in dimeric proteins. However, we did not observe see self-association of AtPRD3 in a yeast two hybrid assay (not shown). Nevertheless, we cannot rule out that self-interaction occurs specifically during meiosis following meiotic specific post-translational modifications of the protein. This is the case for Mer2 phosphorylation which was shown to be necessary for protein-protein interactions with itself and other “DSB proteins” in *S. cerevisiae* [16],[17]. AtPRD2 encodes a putative nuclear protein of 385 aa containing an α-helical coiled-coil structure (Figure 7). The S/T-P-X-X or S/T-S/T-X-X motifs, which are present in many DNA binding proteins [54], are abundant in the N-terminus of AtPRD2 suggesting that AtPRD2 could act on chromosomes to promote DSB formation. Immunolocalisation of AtPRD2 and AtPRD3/AtPAIR1 in wild type and the different context of DSB mutants, as well as an investigation of their physical interaction with other “DSB proteins”, would clarify their roles during meiotic recombination initiation.

No AtPRD2 or AtPRD3 homologues were found outside of plant eukaryote protein databases, suggesting that they are either too divergent to be recognized, or that they are not conserved across kingdoms. Such a degree of divergence was also observed in other organisms. In budding yeast, for example, the majority of “DSB proteins” (Rec102, Rec104, Rec114, Mer2, Mei4) are apparently not conserved in higher eukaryotes. Moreover, even among species closely related to *S. cerevisiae*, most of the “DSB proteins” are weakly conserved, indicating that meiosis specific “DSB proteins” have rapidly diverged even in the *Saccharomyces* genus [2],[61]. Indeed, we observed only a weak level of conservation between AtPRD3 and its rice homologue. Furthermore, we could not identify a homologue within the basal plant *Physcomitrella patens* genome, suggesting that rapid divergence of “DSB proteins“ also occurred inside the plant kingdom. The six “DSB proteins” in *S. pombe* (Mde2, Rec6, Rec15, Rec24, Rec25 and Rec27) have no clear homologues in any other species. By contrast, Spo11 is relatively well conserved among eukaryotes, suggesting that although the catalytic activity of DSB formation is well conserved, the regulation of this process is more divergent.

### AtHOP2/AHP2 and SDS could act with ASY1 to drive DMC1-mediated DSB repair using the homologous chromosome

Our screen also led to the isolation of other asynaptic mutants, including mutations in *DMC1*, *ASY1*, *HOP2* and *SDS*. No DSB formation defects were detected in these mutants but it appears more likely that DSB repair processes are affected. During meiosis the repair machinery is biased toward inter-homolog (IH) events. Rad51 is needed for both inter-homologue (IH) and inter-sister (IS) repair, while Dmc1 seems to be the important player for mediating IH repair [62] which is consistent with the observed phenotype following *DMC1* disruption in *Arabidopsis* [40]. Similarly for ASY1, recent functional data demonstrated its role in coordinating the activity of the RECA homologues to create a bias in favour of IH repair [50]. The most likely explanation is that in the absence of ASY1, most of the DMC1-mediated repair is prevented mostly leading to IS repair [50]. It should be noted, however, that in all the isolated alleles of *asy1*, the CO level was never below 15% of the wild-type level (Table 1), showing that the requirement for ASY1 in IH-repair is not absolute. Interestingly, *Arabidopsis* ASY1 is a HORMA domain protein that exhibits similarity with the *S. cervisiae* Hop1 [44]. In *S. cerevisiae*, the absence of the meiosis specific recombinase Dmc1 leads to cell arrest in prophase due to unrepaired DSBs [63],[64]. However, an efficient repair of the breaks via inter-sister recombination is observed when *Hop1*, *Red1* or *Mek1* is mutated together with *Dmc1* [65]–[68]. Therefore, it has been proposed that these axis proteins, Hop1, Red1 and the protein kinase Mek1, form a complex that mediates the inter-homologue bias in *S. cerevisiae* by suppressing Dmc1-independent strand invasion during meiosis [66],[69],[70].

Our study also identified SDS as a new player of IH-bias in plants. SDS is a plant-specific protein, with a C-terminal region possessing strong similarity with *Arabidopsis* cyclins [42]. Furthermore, it was shown to interact with the *Arabidopsis* CDKs, Cdc2a (CDKA;1) and Cdc2b (CDKB;1), suggesting that SDS is a cyclin that functions with CDKs in the regulation of meiosis [42]. Our study showed that meiotic DSBs are formed in the *sds* background, but are repaired efficiently without IH CO formation, probably using the sister chromatid as a template as is the case for *Atdmc1* or *asy1*. We demonstrated that residual RECA-like foci are formed in an *Atdmc1* context, suggesting that the repair of DSBs via intersister recombination is RAD51-dependent. We also showed that mislocalisation of AtDMC1 (but probably not of AtRAD51) is associated with the *sds* meiotic phenotype. These data suggest that in *Atdmc1*, *sds* and *asy1*, DSBs are repaired via an AtRAD51-dependent pathway using sister chromatids as a template, showing that both SDS and ASY1 are necessary for DMC1-driven interhomologue repair.

Our study revealed that another locus may be involved in IH-bias control: the *Arabidopsis* Hop2 homologue *AHP2* [41]. Interestingly Hop2 together with its partner Mnd1 were proposed to support the activity of the recombinase Dmc1 to promote IH repair [71]. Nevertheless, *hop2* knock-out mutants in most species exhibit meiotic DSB repair defects (similar to mutations in the gene encoding its heterodimeric partner *Mnd1*), suggesting a total absence of DSB repair in these backgrounds. Similarly in *Arabidopsis thaliana* the *ahp2* knock out line shows a total absence of DSB repair [41] whereas the two alleles isolated in this study, probably representing a partial loss of function, appear to be extremely efficient in IS repair. Further investigations will be necessary to understand how this uncoupling of HOP2 function is achieved.

### Conclusive remarks

This study provides, for the first time in a higher eukaryote, a global view of the molecular players involved in meiotic DSB formation on one hand and in the choice of homologous partner for DSB repair on another hand. Their studies will undoubtedly provide important information on how these two steps of meiotic recombination are regulated.

## Materials and Methods

### Plant material

All lines shown in Table 1 were obtained from the Versailles collection of *Arabidopsis* T-DNA transformants (Ws-4 accession) available at http://www-ijpb.versailles.inra.fr/en/sgap/equipes/variabilite/crg/ [43]. The *Atprd2-1* (E19-68 line) and *Atspo11-1-2* (E24 line) mutant lines were isolated from *Arabidopsis* ecotype Columbia (Col-0) as described in [8]. The *Atprd3-3* (line GABI_677DO6), *Atprd3-4* (line GABI_357H01) and *sds-2* (line SAIL_129_F09) mutant lines were obtained from the collection of T-DNA mutants from the Salk Institute Genomic Analysis Laboratory (Col-0 accession) (SIGnAL, http://signal.salk.edu/cgi-bin/tdnaexpress) [72] and provided by NASC (http://nasc.nott.ac.uk/). The *Atdmc1-1*, *Atspo11-1-1*, *asy1-1*, *Atrad51-1* and *Atmre11-3* mutants were described in [40], [8], [50], [47] and [22] respectively.

### Mutation cloning

Linkage between the T-DNA insert (kanamycin resistance marker) and the meiotic phenotype was checked as described in [8] for all Table 1 mutants. When tagged, the mutation was isolated by cloning T-DNA flanking sequences either using TAIL PCR [73] or kanamycin rescue [74]. Untagged mutations were roughly mapped by SNP genotyping as described in Text S1. This rough positioning of the mutation allowed the quick identification of putatively allelic mutations and the identification of candidate genes. For loci which did not colocalise with a putative candidate gene, fine gene mapping was carried out using additional semi-sterile plants that were genotyped for markers in the selected genomic region (see Table S2 for marker list). The microsatellite and indel markers were amplified by PCR and the length polymorphisms were revealed by agarose gel electrophoresis as described by [75]. CAPS markers were PCR amplified and then digested by the suitable restriction enzyme before agarose gel electrophoresis. This fine mapping reduced the candidate intervals to 56, 418 and 371 kb for *AtPRD2*, *AtPRD3* and *AtHOP2* respectively. Then sequencing of the candidate genes was undertaken until mutations were identified. Gene identification was confirmed by sequencing the selected candidate gene in other available alleles recovered by the screen, and by confirming the mutation in T-DNA mutants obtained from NASC (http://nasc.nott.ac.uk/) (see Plant Material section).

### Complementation tests

We tested for allelism between the mutations that mapped to similar locations, by crossing heterozygous plants for each of the mutations. When allelic, a quarter of the F1 plants were semi-sterile.

### Double mutant generation

Double mutants were obtained by crossing plants heterozygous for each of the mutations. The resulting hybrids were self-pollinated. PCR screening was used to select the plants homozygous for both mutations among the semi-sterile plants in the F2 progeny.

### PCR genotyping

See Text S1.

### Sequence analyses

Protein sequence similarity searches were performed at the National Centre for Biotechnology Information (http://www.ncbi.nlm.nih.gov/BLAST/), the Arabidopsis Information Resource (TAIR, http://www.arabidopsis.org/Blast), and the Joint Genome Institute (JGI, http://genome.jgi-psf.org/), using BLOSUM45 matrix and default parameters. Multiple alignments were performed with Bioedit software (http://www.mbio.ncsu.edu/BioEdit/bioedit.html).

*AtPRD2* homologues: predicted *Oryza sativa* PRD2 (OsPRD2) was derived from genomic sequence Os08g0555800 after genescan processing, *Vitis vinifera* VvPRD2 is CAO66652, *Populous trichocarpa* PtPRD2 was obtained from JGI (fgenesh4_pm.C_LG_VI000547), *Physcomitrella patens* PpPRD2 was obtained from JGI (jgi|Phypa1_1|73600|fgenesh1_pg.scaffold_42000158).

*AtPRD3 homologues*: *Oryza sativa* PAIR1 accession number is NP_001048684, *Populous trichocarpa* PtPRD3 is ABK92867

### Microscopy

Comparison of early stages of macrosporogenesis and the development of pollen mother cells was carried out as described in [8]. Preparation of prophase stage spreads for immunocytology was performed according to [76], with the modifications described in [52]. The ASY1 and ZYP1 polyclonal antibodies [76],[77] were used at a working dilution of 1∶500. The AtDMC1 and AtDMC1/AtRAD51 antibodies were described in [49],[51],[52] and used at a working dilution of 1∶20 and 1∶50 respectively. All observations were made using a Leica DMRXA2 microscope; photographs were taken using a CoolSNAP HQ (Roper) camera driven by Open LAB 4.0.4 software; all images were further processed with Open LAB 4.0.4 or Adobe Photoshop 8.0.

## Supporting Information

Figure S1 *Atprd2* and *Atprd3* mutants are sterile. *Atprd2* and *Atprd3* plants (*Atprd2* shown here in B) look like wild-type (A), except that they have shorter siliques (arrows).
(0.64 MB DOC)

Figure S2 Male and female gametophyte development is impaired in *Atprd2* and *Atprd3* mutants. (A–C) Viability of male gametophyte at maturity (pollen grains) after Alexander staining. Cytoplasm from viable pollen grains is coloured purple. Pollen grain cell walls are stained green. Numerous dead pollen grains can be observed in both mutants in comparison to wild-type. (D–E) DIC observation of mature ovules. In a wild type-ovule (D) some of the seven cells of the mature embryo sac can be observed (black arrows) whereas, at the same stage of development no embryo sac has developed in mutants (E–F), and only degenerated cells can be seen (white arrows). (G–I) DIC observation of the product of male meiosis. In wild-type (G), the four meiotic products are observed encased in a callose wall forming a regular tetrad of microspores (three out of the four cells can be seen). In mutants (H–I), irregular tetrads and polyads are observed.
(0.99 MB DOC)

Figure S3 *Atprd2* and *Atprd3* mutants show defective male meiosis. Comparison of DAPI-stained pollen mother cells during meiosis for a wild-type plant (A–E), *Atprd2* (G–K) and *Atprd3* (M–Q). (A,G,M): pachytene or pachytene-like stages, (B, H, N): diakinesis, (C, I, O): metaphase I/anaphase I transition (D, J, P): metaphase II/anaphase II transition, and (E, K, Q): telophase II. Scale bar, 10 µm. (F, L, R) Co-immunolocalisation of ASY1 (red) and ZYP1 (green) in wild-type (F), *Atprd2* (L) and *Atprd3* (R) male meiocytes. For each cell, only the overlay of both signals is shown. Scale bar, 10 µm.
(0.48 MB DOC)

Table S1 Molecular characterisation of cloned mutations.
(0.04 MB XLS)

Table S2 Molecular markers used for fine mapping of mutations in AtPRD2, AtPRD3, and AtHOP2.
(0.02 MB XLS)

Text S1 Supplementary material and methods.
(0.05 MB DOC)

## References

[1] Keeney S (2001). Mechanism and control of meiotic recombination initiation.. Curr Top Dev Biol.

[2] Keeney S (2007). Spo11 and the Formation of DNA Double-Strand Breaks in Meiosis.

[3] Malik SB, Ramesh MA, Hulstrand AM, Logsdon JM (2007). Protist homologs of the meiotic Spo11 gene and topoisomerase VI reveal an evolutionary history of gene duplication and lineage-specific loss.. Mol Biol Evol.

[4] Celerin M, Merino ST, Stone JE, Menzie AM, Zolan ME (2000). Multiple roles of Spo11 in meiotic chromosome behavior.. Embo J.

[5] Dernburg AF, McDonald K, Moulder G, Barstead R, Dresser M (1998). Meiotic recombination in C. elegans initiates by a conserved mechanism and is dispensable for homologous chromosome synapsis.. Cell.

[6] McKim KS, Hayashi-Hagihara A (1998). mei-W68 in Drosophila melanogaster encodes a Spo11 homolog: evidence that the mechanism for initiating meiotic recombination is conserved.. Genes Dev.

[7] Storlazzi A, Tesse S, Gargano S, James F, Kleckner N (2003). Meiotic double-strand breaks at the interface of chromosome movement, chromosome remodeling, and reductional division.. Genes Dev.

[8] Grelon M, Vezon D, Gendrot G, Pelletier G (2001). AtSPO11-1 is necessary for efficient meiotic recombination in plants.. Embo J.

[9] Hartung F, Angelis KJ, Meister A, Schubert I, Melzer M (2002). An archaebacterial topoisomerase homolog not present in other eukaryotes is indispensable for cell proliferation of plants.. Curr Biol.

[10] Hartung F, Puchta H (2000). Molecular characterisation of two paralogous SPO11 homologues in Arabidopsis thaliana.. Nucleic Acids Res.

[11] Hartung F, Puchta H (2001). Molecular characterization of homologues of both subunits A (SPO11) and B of the archaebacterial topoisomerase 6 in plants.. Gene.

[12] Sugimoto-Shirasu K, Stacey NJ, Corsar J, Roberts K, McCann MC (2002). DNA topoisomerase VI is essential for endoreduplication in Arabidopsis.. Curr Biol.

[13] Yin Y, Cheong H, Friedrichsen D, Zhao Y, Hu J (2002). A crucial role for the putative Arabidopsis topoisomerase VI in plant growth and development.. Proc Natl Acad Sci U S A.

[14] Hartung F, Wurz-Wildersinn R, Fuchs J, Schubert I, Suer S (2007). The catalytically active tyrosine residues of both SPO11-1 and SPO11-2 are required for meiotic double-strand break induction in Arabidopsis.. Plant Cell.

[15] Stacey NJ, Kuromori T, Azumi Y, Roberts G, Breuer C (2006). Arabidopsis SPO11-2 functions with SPO11-1 in meiotic recombination.. Plant Journal.

[16] Henderson KA, Kee K, Maleki S, Santini PA, Keeney S (2006). Cyclin-dependent kinase directly regulates initiation of meiotic recombination.. Cell.

[17] Sasanuma H, Hirota K, Fukuda T, Kakusho N, Kugou K (2008). Cdc7-dependent phosphorylation of Mer2 facilitates initiation of yeast meiotic recombination.. Genes Dev.

[18] Wan L, Niu H, Futcher B, Zhang C, Shokat KM (2008). Cdc28-Clb5 (CDK-S) and Cdc7-Dbf4 (DDK) collaborate to initiate meiotic recombination in yeast.. Genes Dev.

[19] Bleuyard JY, Gallego ME, White CI (2004). Meiotic defects in the Arabidopsis rad50 mutant point to conservation of the MRX complex function in early stages of meiotic recombination.. Chromosoma.

[20] Gerecke EE, Zolan ME (2000). An mre11 mutant of Coprinus cinereus has defects in meiotic chromosome pairing, condensation and synapsis.. Genetics.

[21] Merino ST, Cummings WJ, Acharya SN, Zolan ME (2000). Replication-dependent early meiotic requirement for Spo11 and Rad50.. Proc Natl Acad Sci U S A.

[22] Puizina J, Siroky J, Mokros P, Schweizer D, Riha K (2004). Mre11 deficiency in Arabidopsis is associated with chromosomal instability in somatic cells and Spo11-dependent genome fragmentation during meiosis.. Plant Cell.

[23] Waterworth WM, Altun C, Armstrong SJ, Roberts N, Dean PJ (2007). NBS1 is involved in DNA repair and plays a synergistic role with ATM in mediating meiotic homologous recombination in plants.. Plant J.

[24] Young JA, Hyppa RW, Smith GR (2004). Conserved and nonconserved proteins for meiotic DNA breakage and repair in yeasts.. Genetics.

[25] Arora C, Kee K, Maleki S, Keeney S (2004). Antiviral protein Ski8 is a direct partner of Spo11 in meiotic DNA break formation, independent of its cytoplasmic role in RNA metabolism.. Mol Cell.

[26] Evans DH, Li YF, Fox ME, Smith GR (1997). A WD repeat protein, Rec14, essential for meiotic recombination in Schizosaccharomyces pombe.. Genetics.

[27] Jolivet S, Vezon D, Froger N, Mercier R (2006). Non conservation of the meiotic function of the Ski8/Rec103 homolog in Arabidopsis.. Genes Cells.

[28] Tesse S, Storlazzi A, Kleckner N, Gargano S, Zickler D (2003). Localization and roles of Ski8p protein in Sordaria meiosis and delineation of three mechanistically distinct steps of meiotic homolog juxtaposition.. Proc Natl Acad Sci U S A.

[29] De Veaux LC, Hoagland NA, Smith GR (1992). Seventeen complementation groups of mutations decreasing meiotic recombination in Schizosaccharomyces pombe.. Genetics.

[30] Gregan J, Rabitsch PK, Sakem B, Csutak O, Latypov V (2005). Novel genes required for meiotic chromosome segregation are identified by a high-throughput knockout screen in fission yeast.. Curr Biol.

[31] Martin-Castellanos C, Blanco M, Rozalen AE, Perez-Hidalgo L, Garcia AI (2005). A large-scale screen in S. pombe identifies seven novel genes required for critical meiotic events.. Curr Biol.

[32] Ponticelli AS, Smith GR (1989). Meiotic recombination-deficient mutants of Schizosaccharomyces pombe.. Genetics.

[33] McKim KS, Green-Marroquin BL, Sekelsky JJ, Chin G, Steinberg C (1998). Meiotic synapsis in the absence of recombination.. Science.

[34] Sekelsky JJ, McKim KS, Messina L, French RL, Hurley WD (1999). Identification of novel Drosophila meiotic genes recovered in a P-element screen.. Genetics.

[35] Mehrotra S, McKim KS (2006). Temporal analysis of meiotic DNA double-strand break formation and repair in Drosophila females.. PLoS Genet.

[36] De Muyt A, Vezon D, Gendrot G, Gallois JL, Stevens R (2007). AtPRD1 is required for meiotic double strand break formation in Arabidopsis thaliana.. Embo J.

[37] Libby BJ, Reinholdt LG, Schimenti JC (2003). Positional cloning and characterization of Mei1, a vertebrate-specific gene required for normal meiotic chromosome synapsis in mice.. Proc Natl Acad Sci U S A.

[38] Munroe RJ, Bergstrom RA, Zheng QY, Libby B, Smith R (2000). Mouse mutants from chemically mutagenized embryonic stem cells.. Nat Genet.

[39] Libby BJ, De La Fuente R, O'Brien MJ, Wigglesworth K, Cobb J (2002). The mouse meiotic mutation mei1 disrupts chromosome synapsis with sexually dimorphic consequences for meiotic progression.. Dev Biol.

[40] Couteau F, Belzile F, Horlow C, Grandjean O, Vezon D (1999). Random chromosome segregation without meiotic arrest in both male and female meiocytes of a dmc1 mutant of Arabidopsis.. Plant Cell.

[41] Schommer C, Beven A, Lawrenson T, Shaw P, Sablowski R (2003). AHP2 is required for bivalent formation and for segregation of homologous chromosomes in Arabidopsis meiosis.. Plant J.

[42] Azumi Y, Liu D, Zhao D, Li W, Wang G (2002). Homolog interaction during meiotic prophase I in Arabidopsis requires the SOLO DANCERS gene encoding a novel cyclin-like protein.. Embo J.

[43] Bechtold N, Ellis J, Pelletier G (1993). In Planta, Agrobacterium mediated gene transfer by integration of adult Arabidopsis thaliana plants.. C R Acad Sci Paris.

[44] Caryl AP, Armstrong SJ, Jones GH, Franklin FC (2000). A homologue of the yeast HOP1 gene is inactivated in the Arabidopsis meiotic mutant asy1.. Chromosoma.

[45] Vignard J, Siwiec T, Chelysheva L, Vrielynck N, Gonord F (2007). The interplay of RecA-related proteins and the MND1-HOP2 complex during meiosis in Arabidopsis thaliana.. PLoS Genet.

[46] Siaud N, Dray E, Gy I, Gerard E, Takvorian N (2004). Brca2 is involved in meiosis in Arabidopsis thaliana as suggested by its interaction with Dmc1.. Embo J.

[47] Li W, Chen C, Markmann-Mulisch U, Timofejeva L, Schmelzer E (2004). The Arabidopsis AtRAD51 gene is dispensable for vegetative development but required for meiosis.. Proc Natl Acad Sci U S A.

[48] Masson JY, West SC (2001). The Rad51 and Dmc1 recombinases: a non-identical twin relationship.. Trends Biochem Sci.

[49] Chelysheva L, Gendrot G, Vezon D, Doutriaux MP, Mercier R (2007). Zip4/Spo22 is required for class I CO formation but not for synapsis completion in Arabidopsis thaliana.. PLoS Genet.

[50] Sanchez-Moran E, Santos JL, Jones GH, Franklin FC (2007). ASY1 mediates AtDMC1-dependent interhomolog recombination during meiosis in Arabidopsis.. Genes Dev.

[51] Anderson LK, Offenberg HH, Verkuijlen WM, Heyting C (1997). RecA-like proteins are components of early meiotic nodules in lily.. Proc Natl Acad Sci U S A.

[52] Chelysheva L, Diallo S, Vezon D, Gendrot G, Vrielynck N (2005). AtREC8 and AtSCC3 are essential to the monopolar orientation of the kinetochores during meiosis.. J Cell Sci.

[53] Combet C, Blanchet C, Geourjon C, Deleage G (2000). NPS@: network protein sequence analysis.. Trends Biochem Sci.

[54] Suzuki M (1989). SPXX, a frequent sequence motif in gene regulatory proteins.. J Mol Biol.

[55] Nonomura K, Nakano M, Fukuda T, Eiguchi M, Miyao A (2004). The novel gene HOMOLOGOUS PAIRING ABERRATION IN RICE MEIOSIS1 of rice encodes a putative coiled-coil protein required for homologous chromosome pairing in meiosis.. Plant Cell.

[56] Borde V, Lin W, Novikov E, Petrini JH, Lichten M (2004). Association of Mre11p with double-strand break sites during yeast meiosis.. Mol Cell.

[57] Kee K, Protacio RU, Arora C, Keeney S (2004). Spatial organization and dynamics of the association of Rec102 and Rec104 with meiotic chromosomes.. Embo J.

[58] Li J, Hooker GW, Roeder GS (2006). Saccharomyces cerevisiae Mer2, Mei4 and Rec114 form a complex required for meiotic double-strand break formation.. Genetics.

[59] Maleki S, Neale MJ, Arora C, Henderson KA, Keeney S (2007). Interactions between Mei4, Rec114, and other proteins required for meiotic DNA double-strand break formation in Saccharomyces cerevisiae.. Chromosoma.

[60] Cervantes MD, Farah JA, Smith GR (2000). Meiotic DNA breaks associated with recombination in S. pombe.. Mol Cell.

[61] Richard GF, Kerrest A, Lafontaine I, Dujon B (2005). Comparative genomics of hemiascomycete yeasts: genes involved in DNA replication, repair, and recombination.. Mol Biol Evol.

[62] Pâques F, Haber JE (1999). Multiple pathways of recombination induced by double-strand breaks in *Saccharomyces cerevisiae*.. Microbiol Mol Biol Rev.

[63] Bishop DK, Park D, Xu L, Kleckner N (1992). DMC1: a meiosis-specific yeast homolog of E. coli recA required for recombination, synaptonemal complex formation, and cell cycle progression.. Cell.

[64] Hunter N, Kleckner N (2001). The single-end invasion: an asymmetric intermediate at the double-strand break to double-holliday junction transition of meiotic recombination.. Cell.

[65] Carballo JA, Johnson AL, Sedgwick SG, Cha RS (2008). Phosphorylation of the axial element protein Hop1 by Mec1/Tel1 ensures meiotic interhomolog recombination.. Cell.

[66] Niu H, Wan L, Baumgartner B, Schaefer D, Loidl J (2005). Partner choice during meiosis is regulated by Hop1-promoted dimerization of Mek1.. Mol Biol Cell.

[67] Schwacha A, Kleckner N (1994). Identification of joint molecules that form frequently between homologs but rarely between sister chromatids during yeast meiosis.. Cell.

[68] Wan L, de los Santos T, Zhang C, Shokat K, Hollingsworth NM (2004). Mek1 kinase activity functions downstream of RED1 in the regulation of meiotic double strand break repair in budding yeast.. Mol Biol Cell.

[69] Niu H, Li X, Job E, Park C, Moazed D (2007). Mek1 kinase is regulated to suppress double-strand break repair between sister chromatids during budding yeast meiosis.. Mol Cell Biol.

[70] Schwacha A, Kleckner N (1997). Interhomolog bias during meiotic recombination: meiotic functions promote a highly differentiated interhomolog-only pathway.. Cell.

[71] Chen YK, Leng CH, Olivares H, Lee MH, Chang YC (2004). Heterodimeric complexes of Hop2 and Mnd1 function with Dmc1 to promote meiotic homolog juxtaposition and strand assimilation.. Proc Natl Acad Sci U S A.

[72] Alonso JM, Stepanova AN, Leisse TJ, Kim CJ, Chen H (2003). Genome-wide insertional mutagenesis of Arabidopsis thaliana.. Science.

[73] Liu YG, Mitsukawa N, Oosumi T, Whittier RF (1995). Efficient isolation and mapping of Arabidopsis thaliana T-DNA insert junctions by thermal asymmetric interlaced PCR.. Plant J.

[74] Bouchez D, Vittorioso P, Courtial B, Camilleri C (1996). Kanamycin rescue : a simple technique for the recovery of T-DNA flanking sequences.. Plant Molecular Biology reporter.

[75] Loudet O, Chaillou S, Camilleri C, Bouchez D, Daniel-Vedele F (2002). Bay-0 x Shahdara recombinant inbred line population: a powerful tool for the genetic dissection of complex traits in Arabidopsis.. Theor Appl Genet.

[76] Armstrong SJ, Caryl AP, Jones GH, Franklin FCH (2002). Asy1, a protein required for melotic chromosome synapsis, localizes to axis-associated chromatin in Arabidopsis and Brassica.. J Cell Sci.

[77] Higgins JD, Armstrong SJ, Franklin FC, Jones GH (2004). The Arabidopsis MutS homolog AtMSH4 functions at an early step in recombination: evidence for two classes of recombination in Arabidopsis.. Genes Dev.

