# Supplementary figures and images for: A High Throughput Genetic Screen Identifies New Early Meiotic Recombination Functions in Arabidopsis thaliana
Source: PLoS Genet. 2009 Sep 18;5(9):e1000654. doi: 10.1371/journal.pgen.1000654 (PMC2735182; doi:10.1371/journal.pgen.1000654)

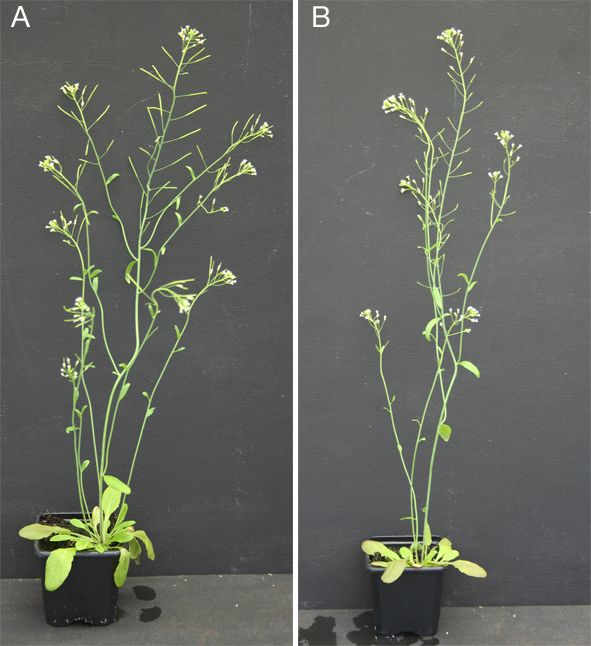

Supplement: Figure S1 — Atprd2 and Atprd3 mutants are sterile. Atprd2 and Atprd3 plants (Atprd2 shown here in B) look like wild-type (A), except that they have shorter siliques (arrows). (0.64 MB DOC) [file pgen.1000654.s001.doc]

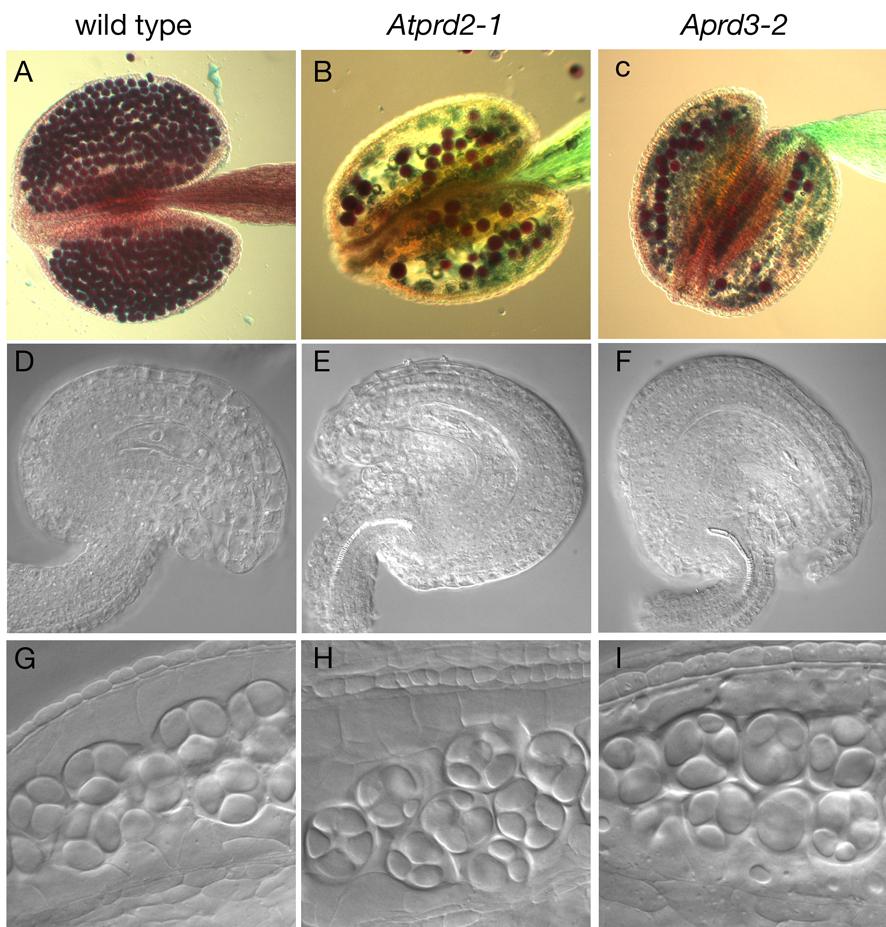

Supplement: Figure S2 — Male and female gametophyte development is impaired in Atprd2 and Atprd3 mutants. (A–C) Viability of male gametophyte at maturity (pollen grains) after Alexander staining. Cytoplasm from viable pollen grains is coloured purple. Pollen grain cell walls are stained green. Numerous dead pollen grains can be observed in both mutants in comparison to wild-type. (D–E) DIC observation of mature ovules. In a wild type-ovule (D) some of the seven cells of the mature embryo sac can be observed (black arrows) whereas, at the same stage of development no embryo sac has developed in mutants (E–F), and only degenerated cells can be seen (white arrows). (G–I) DIC observation of the product of male meiosis. In wild-type (G), the four meiotic products are observed encased in a callose wall forming a regular tetrad of microspores (three out of the four cells can be seen). In mutants (H–I), irregular tetrads and polyads are observed. (0.99 MB DOC) [file pgen.1000654.s002.doc]

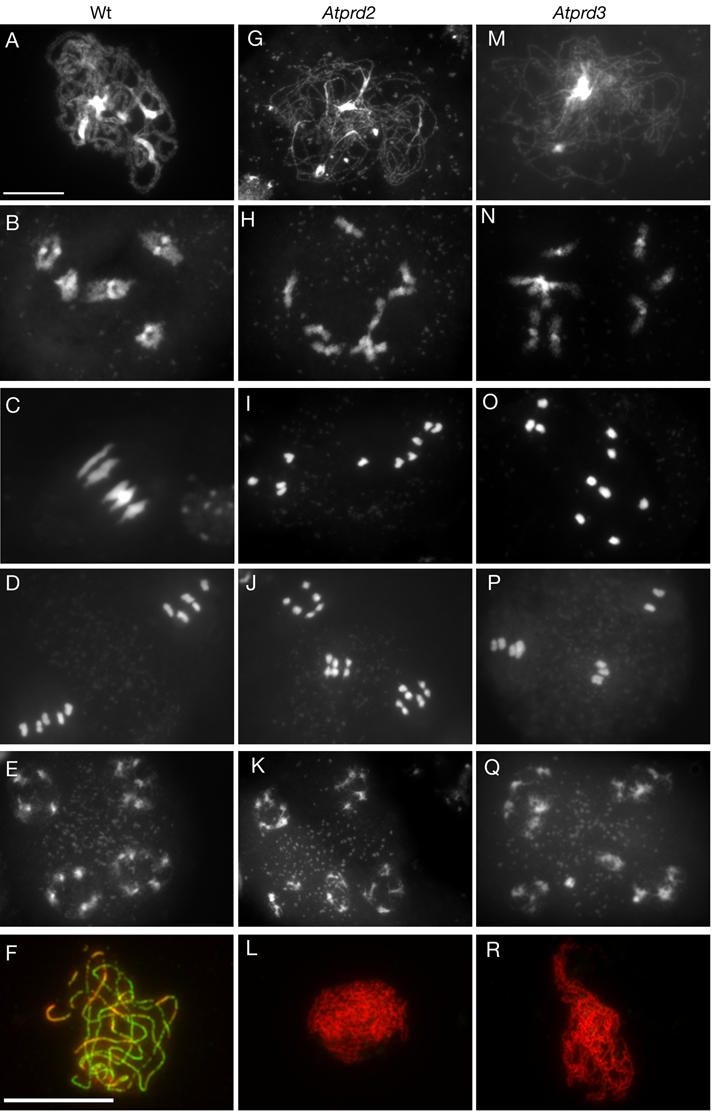

Supplement: Figure S3 — Atprd2 and Atprd3 mutants show defective male meiosis. Comparison of DAPI-stained pollen mother cells during meiosis for a wild-type plant (A–E), Atprd2 (G–K) and Atprd3 (M–Q). (A,G,M): pachytene or pachytene-like stages, (B, H, N): diakinesis, (C, I, O): metaphase I/anaphase I transition (D, J, P): metaphase II/anaphase II transition, and (E, K, Q): telophase II. Scale bar, 10 µm. (F, L, R) Co-immunolocalisation of ASY1 (red) and ZYP1 (green) in wild-type (F), Atprd2 (L) and Atprd3 (R) male meiocytes. For each cell, only the overlay of both signals is shown. Scale bar, 10 µm. (0.48 MB DOC) [file pgen.1000654.s003.doc]
