# Supplementary material for: A High Throughput Genetic Screen Identifies New Early Meiotic Recombination Functions in Arabidopsis thaliana
Source: PLoS Genet. 2009 Sep 18;5(9):e1000654. doi: 10.1371/journal.pgen.1000654 (PMC2735182; doi:10.1371/journal.pgen.1000654)
Supplement: Text S1 — Supplementary material and methods. (0.05 MB DOC) [file pgen.1000654.s006.doc]

**Supplementary** **Material and Methods:**

**I-SNP genotyping**

For each untagged line, a semi-sterile mutant plant (Ws-4 background) was crossed to wild-type Col-0 accession. For each F2 population, 31 mutant plants were selected and DNA was extracted as described in [1]. 48 SNP markers were selected that were evenly distributed along the chromosomes and polymorphic between Col-0 and Ws-4 (see the table below). The 48 SNP markers were multiplexed in one set, and genotyped using the SNPlex technology (Applied Biosystems, Foster City, CA) according to the supplier’s protocols. In the F2 population, all markers segregated 50:50 Col-0:Ws-4, but because only mutant plants were selected for mapping, markers linked to the investigated mutation were enriched in Ws-4 allele. This distortion of segregation allowed the rough positioning of the mutation as well as the quick identification of putatively allelic mutations. These were then tested by direct complementation tests or by candidate gene sequencing.

**SNP markers used in this study**

| Marker | Sequence |
| --- | --- |
| c1_02992 | TTTCATGTACGTTTG[A/G]TTATACACGTAAAAG |
| c1_05593 | CGTTTTAGCTTTCCC[C/G]AGTTTACTTTCTTAT |
| c1_08385 | AAGTTTTTGATAGAC[G/A]AAAATTACGGATTTA |
| c1_12295 | TGTGAACCCTTGTCT[A/C]ACCTCTGTTGGATGG |
| c1_13926 | CGTCATTCTAAGCAC[G/C]AGATTCATGATGCAA |
| c1_15634 | AGAAAGGAATAAATT[T/G]TGATTCATCTTGTGG |
| c1_18433 | AAACCCGACCCGACT[A/C]GGATCCGACGCAACA |
| c1_20384 | CAATTTTTTTGTTTT[C/G]TTTTGTGTATGTAAC |
| c1_22181 | GACATTATGAATCAT[G/A]GAAAATTTGGAGATA |
| c1_23381 | ACACATGACTACATA[T/A]ACAAAATCACAAAAT |
| c1_25698 | GTTATTGGTAGGTGA[G/A]ATTTGTTTATCAAGA |
| *c1_27867 | GAATCAACAGAGTTC[G/A]AGAAAAATCAAAAAC |
| c2_00593 | AGATTCTTGTCCCAC[T/C]AAACCAAGTCCAACT |
| c2_02365 | GAGATTTAAAAACCA[T/C]ATAATCAGTGGACTT |
| c2_04263 | CCTTGCCGACCATTG[T/G]AGACTCTCTTCAAGA |
| c2_07650 | TCAGATTTTGAAGAT[G/A]CGAAAACAAATCTGG |
| c2_10250 | GTTTGAGATTACTGA[C/A]ACTGAGAACTTCATA |
| c2_13472 | CAAGGTTTACTCTGT[T/C]TAGTGTCCGGATTCT |
| c2_15252 | TTTCAGGAGGGTTTA[C/T]AACTTGAGGCACAGA |
| c2_16837 | ACATGAAAGCTCAAA[T/C]CAATTTCTATTCAAC |
| c2_18753 | AATCTAGAGAAGTTT[A/T]TGTTCTGATTCAACA |
| c3_00885 | TTTTCGTATGAATCG[G/A]AATCAAGTTAGTTAA |
| c3_02968 | ATTGCTGAGAAACGC[G/T]TAAGCAGACATAATG |
| c3_05141 | TGAAAACAAACCCCT[A/T]CTTAATACTTTGTAT |
| c3_08042 | TCGGTGTTGACATCC[A/T]ACAAAATGCTTAGAT |
| c3_11192 | GTAATATTGTGACTT[C/T]GTGAAAATTCCACTG |
| c3_14097 | GCCAAGTTTCTTGAT[A/G]TCTTGCCTGATCCAA |
| c3_16677 | AGAAAAACGGTGTGG[G/A]CCATATTCTTTTTCC |
| c3_18180 | TTTCAGATAATGTTT[T/G]CAGGTCTTTGATTTC |
| c3_22147 | TGGAGAATCAACTTC[C/T]CTTAATCTCAATCAG |
| c3_20729 | CTTCGCCATCTCTAC[T/C]AAGCCCTTTGACCTA |
| *c4_01607 | CCTCCATTTCGTTTA[A/G]TTCGTGAGCTTTCCT |
| c4_03833 | AATGATGGAAAAATT[A/G]CACTTGCGGTGGTAA |
| c4_06923 | GATCAAGATTAAAGT[G/A]AATTATGTTGTCCAG |
| c4_08930 | CTATATAGTTGTGTT[A/T]GGTTTAGGAATTTCC |
| c4_11878 | CTAACGATTATGTAA[T/C]ACAACAAGAGCCTTG |
| c4_14819 | GTGAATGCTCTGTTT[A/C]TAGCTTGTCTATTAT |
| c4_17684 | TTGGAGAATCCAAAT[T/G]ACGATGAATTGGTAA |
| c5_00576 | CTAAGAAATCTTAGT[A/T]CTTCTTTCTTCATTC |
| c5_04011 | AGGGGACTACAAGGC[C/T]TTTCTTCTAGCTTTG |
| c5_06820 | CAGAAGTTGGTACCC[T/C]GACTGTGCCTACACA |
| c5_08563 | TAGGTGAATATAAAC[T/G]AAGAGATCGAAAGTG |
| c5_10428 | GTGATTGTATGACGG[A/G]AAGCATTGTGTGCAA |
| c5_13614 | CATGGAATGCTAAAA[G/C]TCCTCCTTGCACTTG |
| c5_16368 | GTCTTAAACAGCATG[T/A]AGTGCGTCCAAGGAA |
| c5_19316 | TGACATGTCCCTGAA[A/C]TCTGCCTTTGTCTCA |
| c5_22415 | CAAACGCGGCGTATT[T/A]ATGTGATATGGCTTC |
| c5_24997 | TGAGATCACGAGCCA[C/T]CTCGGAGCAGAAATC |

List of the 48 SNP markers used in this study. All but the two marked by an asterisk were defined in [1]. The marker names give the number of the chromosome followed by the physical position in kb of the markers on the chromosome (TAIR 7.0 Col-0 genomic sequence, April 23, 2007, http://www.arabidopsis.org). The SNPs are in brackets, the first allele is the Col-0 one. The second is the Ws one.

1. Simon, M., et al.*, Quantitative trait loci mapping in five new large recombinant inbred line populations of Arabidopsis thaliana genotyped with consensus single-nucleotide polymorphism marker*s. Genetics, 2008**. 1**78(4): p. 2253-64.

##### II-PCR genotyping

| *Allele* | Primers used for wild-type allele | Primers used for mutant allele |
| --- | --- | --- |
| *Atmre11-3* | MRE11-1 and MRE11-3 | MRE11-3 and LBc-1 |
| *Atrad51-1* | RAD51-1-1 and RAD51-1-2 | RAD51-1-1 and LbGabi1 |
| *Atdmc1-1* | WTF_DMC1 and WTR_DMC1 | DMC1-1 and LBFeldman |
| *Atspo11-1-1* | MG52 and MG96 | MG52 and LbBar2 |
| *sds-2* | SDS-P9 and SDS P10 | SDS-P9 and Lbsail3 |
| *asy1-2* | ASY1-P1 and ASY1-P2 | ASY1-ex1 and TAG3 |
| *ahp2-2* | N6360002U and N6360002L: 900 bp | N6360002U and N6360002L: 750 bp |
| *Atprd2-1* | pPRD2_5 and pPRD2_6 digested by BclI: 1000pb+360pb | pPRD2_5 and pPRD2_6 digested by BclI: 1000pb+230pb+150pb. |
| *Atprd3-1* | pPRD3-P3 and pPRD3-P4 digested by BseMII.  Allele Ws: 831pb+439pb+150+22+16+7  Allele Col-0: 554+438+231+121pb | pPRD3-P3 and pPRD3-P4 digested by BseMII.  Mutant allele: 982pb+439+ 37+22+16+7. |

## **Primer sequences**

| pPRD2_5 | ATAGATTTTCTCCTGGAACTG |
| --- | --- |
| pPRD2_6 | AGTAGATGCTAACCTGGAAAG |
| pPRD3-P3 | GGTTTCAGTTCTCTCACTCT |
| pPRD3-P4 | CTGCTGAAGTGAAGTGTCTTG |
| MRE11-1 | CCAATGGATGAGGCCTGAAGTT |
| MRE11-3 | GTCTGCCACCACCATAACAT |
| LBc-1 | TGGACCGCTTGCTGCAACTCT |
| RAD51-1-1 | GGTTCCATCACGGAGTTATATGG |
| RAD51-2-1 | AGCCATGATATTCCCACCAATC |
| LbGabi | CCCATTTGGACGTGAATGTAGACAC |
| WTF_DMC1 | CAGAGGCATGAGATGAGATTTTACT |
| WTR_DMC1 | GATTTGGTTAAAAGGATATGGCTTC |
| DMC1-1 | ATGCATACCAAGAAGGTATTAACCA |
| LBFeldman | AAGTTGTCTAAGCGTCAATTTGTTT |
| MG52 | GGATCGGGCCTAAAAGCCAACG |
| MG96 | CTTTGAATGCTGATGGATGCATGTAGTAG |
| LbBar2 | CGTGTGCCAGGTGCCCACGGAATAG |
| SDS-P9 | CTGCTCCCTGATTACAAGCAG |
| SDS-P10 | CTTAACGCATTCAGGCAACTC |
| Lbsail3 | TAGCATCTGAATTTCATAACCAATCTCGATACAC |
| ASY1-P1 | TGGGTTGGGCTGTAACATTA |
| ASY1-P2 | CTAAAGCAGGAACCGATTTA |
| ASY1-ex1 | CAGAAGAAGCGAGTCCTGCT |
| TAG3 | CTG ATA CCA GAC GTT GCC CGC ATA A |
| N6360002U | CACCATCAATACAAAAGACG |
| N6360002L | CCAAGTGATTAAATTCCCAG |
